# Global research trends in programmed cell death in rheumatoid arthritis from 2001 to 2025: a bibliometric analysis

**DOI:** 10.3389/fimmu.2026.1837734

**Published:** 2026-06-10

**Authors:** Bo Wang, Yin Lian, Lina Gao, Zhixin Chen, Chunzhu Gong, Renxuan Yang, Dandan Li

**Affiliations:** 1 Shenzhen Pingle Orthopedics Hospital (Shenzhen Pingshan Traditional Chinese Medicine Hospital), Shenzhen, China; 2 Shenzhen Orthopedics Hospital Affiliated to Guangzhou University of Chinese Medicine, Shenzhen, China; 3 Department of Clinical Medical Research Center, The Second Clinical Medical College of Jinan University (Shenzhen People’s Hospital), Shenzhen, China

**Keywords:** bibliometric, ferroptosis, programmed cell death, pyroptosis, rheumatoid arthritis

## Abstract

**Objective:**

Programmed cell death (PCD) has become an important framework for understanding the pathogenesis of rheumatoid arthritis (RA), but the overall research landscape, intellectual base, and evolving hotspots in this field remain unclear. This study aimed to investigate the global trends, knowledge structure, thematic evolution, and disciplinary diffusion of PCD research in RA through bibliometric analysis.

**Methods:**

Publications on PCD in RA from 2001 to 2025 were retrieved from the Web of Science Core Collection and PubMed. After merging, deduplication, and screening, 3, 168 English-language articles and reviews were included. Bibliometric analyses were performed using Bibliometrix and VOSviewer, with sensitivity analyses conducted to assess the robustness of the main network findings. Annual publication trends, contributions of countries, institutions, authors, and journals, collaboration networks, reference co-citation, author keyword co-occurrence, temporal keyword characteristics, journal bibliographic coupling, and dual-map overlay were analyzed.

**Results:**

A total of 3, 168 publications were identified, and annual publication output showed a clear upward trend, with a notable increase during the later years of the study period. China, the United States, and Japan were the leading contributing countries. Anhui Medical University was the most productive institution, and Zhang Y was the most productive author. Reference co-citation analysis identified six major clusters, showing that the intellectual base of the field remains rooted in classical RA pathogenesis while expanding toward emerging PCD-related mechanisms. Author keyword analysis revealed five major thematic domains. Temporal analysis showed that apoptosis- and fibroblast-like-synoviocyte-related studies remained persistent core topics, whereas pyroptosis, ferroptosis, oxidative stress, and reactive oxygen species gained increasing prominence in recent years. Source and dual-map overlay analyses further indicated that this field mainly spans rheumatology, immunology, molecular biology, and clinical medicine.

**Conclusion:**

Research on PCD in RA has expanded continuously and evolved from an apoptosis-centered framework toward a broader and more differentiated mechanistic landscape. These findings provide a systematic reference for future mechanistic and translational research, while the roles of emerging PCD pathways require further disease-specific validation.

## 1 Introduction

Rheumatoid arthritis (RA) is a chronic autoimmune disease characterized by persistent synovial inflammation, progressive cartilage destruction, and bone erosion, ultimately leading to joint deformity and functional disability (1). In 2020, rheumatoid arthritis affected an estimated 17.6 million people worldwide, with an age-standardized global prevalence of 208.8 cases per 100, 000 population, representing a substantial global health burden (2). Although significant progress has been achieved in the development of disease-modifying antirheumatic drugs (DMARDs) and biologic therapies, a considerable proportion of patients continue to experience incomplete remission or disease relapse (3). Therefore, a deeper understanding of the molecular mechanisms underlying RA pathogenesis remains essential for improving disease management and developing novel therapeutic strategies.

Programmed cell death (PCD) refers to a group of genetically regulated cellular processes that play critical roles in maintaining tissue homeostasis, regulating immune responses, and controlling inflammation (4). Over the past two decades, accumulating evidence has demonstrated that dysregulation of multiple forms of PCD contributes significantly to the initiation and progression of RA (5, 6). Traditionally, apoptosis was considered the primary form of regulated cell death associated with RA pathogenesis (6). Resistance to apoptosis in fibroblast-like synoviocytes (FLS) has been shown to promote synovial hyperplasia and pannus formation, which are hallmark pathological features of RA (7, 8). However, recent studies have revealed that other forms of PCD—including pyroptosis, necroptosis, ferroptosis, and autophagy-related cell death—also play important roles in the inflammatory microenvironment of RA (5, 9).

For example, inflammasome-mediated pyroptosis can promote the release of pro-inflammatory cytokines such as interleukin-1β and interleukin-18, thereby amplifying synovial inflammation (10). Ferroptosis, a form of iron-dependent lipid peroxidation-driven cell death, has been implicated in oxidative stress-related joint damage and inflammatory responses (11, 12). Necroptosis, another regulated necrotic pathway, may contribute to the propagation of inflammatory signaling in synovial tissues (13). These diverse forms of programmed cell death interact with immune cells, synovial fibroblasts, and inflammatory mediators, collectively shaping the pathophysiological processes of RA (5). Consequently, programmed cell death has emerged as an important research focus and a potential therapeutic target in RA.

In recent years, research on programmed cell death in RA has broadened considerably, alongside advances in molecular biology, immunology, and cell death signaling. A growing number of studies have investigated the molecular mechanisms, signaling networks, and potential therapeutic implications of various PCD pathways in RA (9, 14). However, despite the expanding body of literature, the overall development trajectory, knowledge base, and emerging research hotspots in this field remain unclear. Traditional narrative reviews often focus on specific mechanisms or signaling pathways but may not provide a comprehensive overview of the global research landscape. Bibliometric analysis is a quantitative method that applies statistical and mathematical techniques to analyze scientific publications. By examining publication outputs, citation patterns, collaboration networks, and keyword co-occurrence relationships, bibliometric analysis can reveal the intellectual structure, influential contributors, and evolving trends within a research field (15). In recent years, bibliometric approaches have been widely used to evaluate research developments in various biomedical domains, including autoimmune diseases, inflammatory signaling pathways, and different forms of programmed cell death (16–18).

Therefore, the present study aimed to conduct a bibliometric analysis of publications related to programmed cell death in rheumatoid arthritis from 2001 to 2025. By analyzing literature retrieved from the Web of Science Core Collection and PubMed and applying visualization tools such as Bibliometrix and VOSviewer, this study sought to (1) characterize the temporal evolution of publications, (2) identify the most productive countries, institutions, authors, and journals, (3) map collaboration networks, and (4) explore major research hotspots and emerging topics in this rapidly evolving research domain. Beyond descriptive mapping, we further examined how emerging PCD-related themes were structurally connected with the established RA knowledge base.

## 2 Methodology

### 2.1 Data sources and search strategy

Publications on programmed cell death in rheumatoid arthritis (RA) were retrieved from the Web of Science Core Collection (WoSCC) and PubMed. The search covered the period from January 1, 2001 to December 31, 2025. Both databases were searched on the same date to minimize discrepancies caused by database updates.

The search strategy combined terms related to RA with terms representing programmed cell death and its major subtypes. In WoSCC, the search was performed in the Topic field (TS), which covers title, abstract, author keywords, and Keywords Plus. The WoSCC query was as follows:

TS=((“rheumatoid arthritis” OR “arthritis, rheumatoid” OR rheumatoid arthrit*) AND (“programmed cell death” OR “regulated cell death” OR apoptos* OR pyroptos* OR ferroptos* OR necroptos* OR “programmed necrosis” OR “autophagic cell death” OR “autophagy-dependent cell death” OR “autophagy dependent cell death” OR autosis OR anoikis OR entosis OR parthanatos OR NETosis OR “neutrophil extracellular trap*” OR cuproptos* OR disulfidptos* OR oxeiptosis OR alkaliptosis OR PANoptosis OR panoptos* OR “immunogenic cell death” OR “lysosome-dependent cell death” OR “lysosomal cell death” OR “mitochondrial transmembrane permeability-driven necrosis” OR “mitochondrial permeability transition-driven necrosis”)).

Using this strategy, WoSCC initially returned 6, 934 records. After restricting the publication period to 2001–2025, language to English, and document types to articles and reviews, 6, 265 records were retained.

In PubMed, the search combined Medical Subject Headings (MeSH) and free-text terms in titles and abstracts. The query was as follows:

(“Arthritis, Rheumatoid”[Mesh] OR “rheumatoid arthritis”[tiab] OR rheumatoid arthrit*[tiab]) AND (“Regulated Cell Death”[Mesh] OR “Cell Death”[Mesh] OR “programmed cell death”[tiab] OR “regulated cell death”[tiab] OR “Apoptosis”[Mesh] OR apoptos*[tiab] OR “Pyroptosis”[Mesh] OR pyroptos*[tiab] OR “Ferroptosis”[Mesh] OR ferroptos*[tiab] OR “Necroptosis”[Mesh] OR necroptos*[tiab] OR “programmed necrosis”[tiab] OR “Autophagic Cell Death”[Mesh] OR “Autophagy”[Mesh] OR “autophagic cell death”[tiab] OR “autophagy-dependent cell death”[tiab] OR “autophagy dependent cell death”[tiab] OR autosis[tiab] OR anoikis[tiab] OR entosis[tiab] OR parthanatos[tiab] OR NETosis[tiab] OR “neutrophil extracellular trap*”[tiab] OR “Cuproptosis”[Mesh] OR cuproptos*[tiab] OR disulfidptos*[tiab] OR oxeiptosis[tiab] OR alkaliptosis[tiab] OR PANoptosis[tiab] OR panoptos*[tiab] OR “immunogenic cell death”[tiab] OR “lysosome-dependent cell death”[tiab] OR “lysosomal cell death”[tiab] OR “mitochondrial transmembrane permeability-driven necrosis”[tiab]).

PubMed initially returned 4, 605 records. After restricting the publication period to 2001–2025 and language to English, 4, 152 records were retained. WoSCC served as the primary bibliometric data source because it provides relatively complete and standardized bibliographic information, including citation data and journal-based subject categories. PubMed was included mainly to improve biomedical topic coverage. Because PubMed records are not directly equivalent to WoSCC records in terms of bibliometric structuring, subject-related and citation-related standardization was primarily based on WoSCC metadata or matched WoSCC entries after merging. For example, WoSCC records were preferentially used for analyses involving citation relationships, source journals, and subject categories, whereas PubMed records mainly contributed additional biomedically relevant studies to the final screened dataset.

### 2.2 Literature screening and data collection

Records retrieved from WoSCC and PubMed were exported in plain-text or compatible bibliographic formats and imported into R using the bibliometrix package (R version 4.5.1). The two datasets were merged, and duplicate records were identified primarily on the basis of Digital Object Identifiers (DOIs), supplemented by title-based matching when DOI information was unavailable or incomplete. Candidate duplicate pairs were further checked manually when necessary to avoid erroneous removal of non-identical records.

After database merging and deduplication, 2, 961 duplicate records were removed and 7, 456 unique records were retained. During deduplication, WoSCC records were preferentially retained when the same publication was indexed in both WoSCC and PubMed. To improve feasibility and reproducibility, the deduplicated dataset was first refined using predefined bibliographic filters, including language, document type, and topic relevance as reflected in titles, abstracts, and author keywords. Records that were clearly unrelated to rheumatoid arthritis or programmed cell death were excluded at this stage. The remaining records were then independently reviewed by two investigators, and full texts or publisher records were consulted only when bibliographic information was insufficient to determine eligibility. Disagreements were resolved by discussion and, when required, adjudication by a third reviewer.

**Figure 1:**
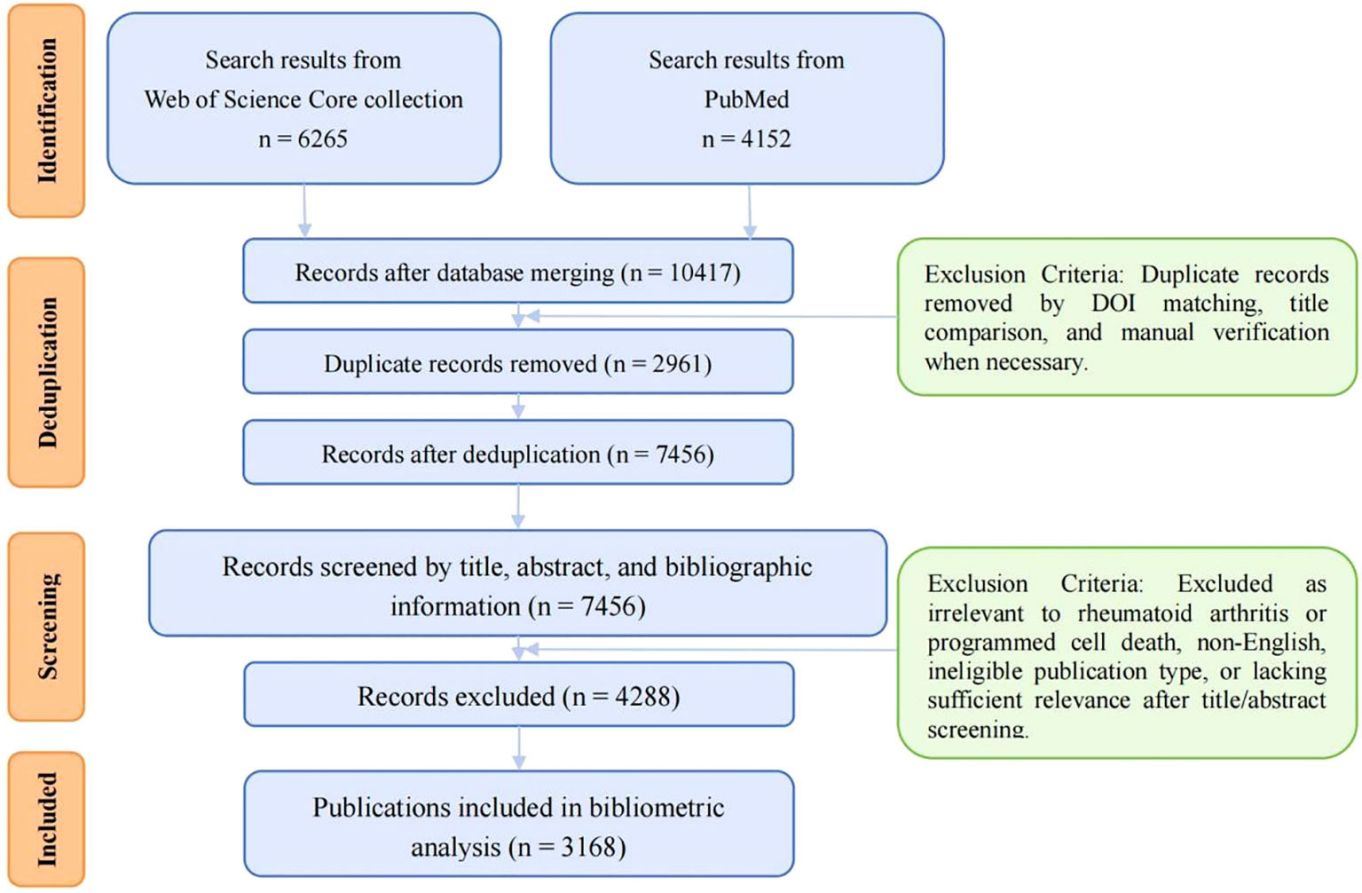
Literature identification and screening flowchart. The search covered publications from January 1, 2001 to December 31, 2025. In WoSCC, the search was performed in the Topic field (TS), which covers titles, abstracts, author keywords, and Keywords Plus. In PubMed, the search combined MeSH terms and free-text terms in titles and abstracts. WoSCC records were restricted to articles and reviews during retrieval, whereas document-type eligibility for PubMed records was further verified during the screening stage.

After screening, 4, 288 records were excluded and 3, 168 publications were finally included in the bibliometric analysis, including 3, 161 WoSCC records and 7 PubMed-only records. Cited-reference information for PubMed-only records was retrieved through DOI/PMID-based matching to external open metadata sources, primarily Crossref/XREF, and converted into a WoSCC-like structure using R before bibliometric processing. Because this study was designed as a bibliometric analysis rather than a conventional systematic review, the literature identification and screening process was documented using a PRISMA 2020-style flow diagram to improve reporting transparency, rather than as a formal PRISMA-compliant evidence-synthesis workflow. The literature identification and screening process is shown in **Figure 1**.

### 2.3 Bibliometric analysis

Bibliometric analysis was performed using R (version 4.5.1) with the bibliometrix package, and network visualization was conducted using VOSviewer (version 1.6.20). The analysis included annual publication trends, contribution analysis of countries, institutions, authors, and journals, collaboration network analysis, author keyword co-occurrence analysis, reference co-citation analysis, journal bibliographic coupling analysis, and dual-map overlay analysis of journals. To further characterize country-level collaboration, we calculated overall publications, total citations, average citations, single-country publications (SCP), multiple-country publications (MCP), and MCP ratio using the same country extraction and counting rule as the country productivity analysis.

In VOSviewer-based network maps, nodes represent countries, institutions, authors, references, keywords, or journals depending on the analytical unit. Node size reflects publication output, occurrence frequency, citation frequency, or total link strength, as appropriate. Links indicate collaborative, co-occurrence, co-citation, or bibliographic coupling relationships, and thicker links represent stronger relationships. In the main analyses, the minimum occurrence threshold was set at 5 for author keyword co-occurrence, and the minimum citation threshold was set at 20 for reference co-citation. For both networks, the clustering resolution was set to 1, the minimum cluster size was set to 10, and the option to merge small clusters was not selected. For institutional and author collaboration networks, the minimum occurrence threshold was set at 5. For journal bibliographic coupling, the minimum threshold was set at 5 documents per source. Before author keyword co-occurrence analysis, author keywords were standardized using a manually curated thesaurus file. The thesaurus was used to harmonize spelling variants, singular and plural forms, abbreviations, and clearly equivalent terms. Broader umbrella terms were not automatically merged with more specific disease entities unless they were exact synonyms.

To assess the robustness of the main network findings, sensitivity analyses were conducted for author keyword co-occurrence, reference co-citation, and dataset source. For author keyword co-occurrence, alternative minimum occurrence thresholds of 4 and 6 were tested against the main threshold of 5. For reference co-citation, alternative minimum citation thresholds of 18 and 22 were tested against the main threshold of 20. All clustering settings were kept unchanged. To assess the influence of PubMed-only records, the seven PubMed-only records were excluded and the author keyword co-occurrence and reference co-citation analyses were repeated using the same settings.

## 3 Results

### 3.1 Temporal trends, major contributors, and global collaboration patterns

#### 3.1.1 Annual publication and citation trends

**Figure 2:**
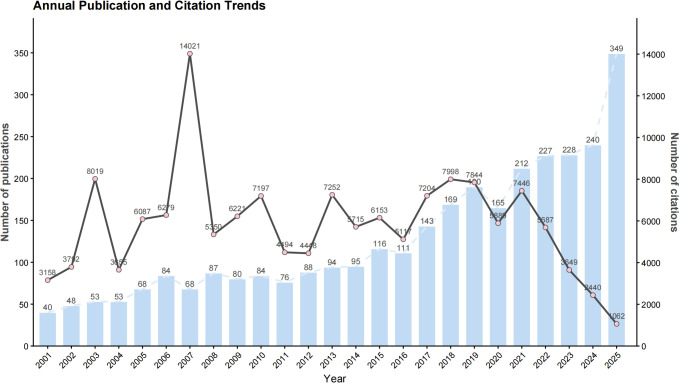
Annual publication and citation trends. Bars represent annual publication output, and the dashed line indicates the overall publication trend. The solid line with circular markers represents annual citations, and the numeric labels above the markers indicate citation counts. The left y-axis corresponds to publications and the right y-axis to citations. The terminal year should be interpreted cautiously because indexing updates and citation accumulation may not have been fully complete.

The temporal distribution of publications and citations in this research field is shown in **Figure 2**. Overall, the number of publications exhibited a clear upward trajectory over time, indicating a continuous expansion of research activity. In the early years, the annual publication output remained relatively low and stable, reflecting the exploratory nature of the field during its initial phase. Subsequently, the number of publications increased steadily and showed a marked acceleration in recent years, suggesting growing academic attention and research investment. Because 2025 was the terminal year of the search window, publication output and citation counts for this year should be interpreted cautiously, as database indexing and citation accumulation may not have been fully complete.

A similar pattern was observed for the annual citation counts. Although citations were limited in the early stage due to the small number of available publications, citation frequency gradually increased as the field accumulated more research outputs. In later years, the annual citation counts rose substantially, indicating that the existing body of literature has gained increasing scholarly recognition and influence.

Based on the evolution of publication output and citation impact, the development of this research field can be broadly divided into two stages: an early exploratory stage (2001–2016) and a rapid development stage (2017–2025). The early stage was characterized by relatively few publications and modest citation activity, reflecting the initial exploration and conceptual development of the topic. In contrast, the rapid development stage was marked by a pronounced increase in both publication output and citation counts, indicating the emergence of sustained research interest and the formation of a more mature knowledge base.

#### 3.1.2 Global research contributions and collaboration patterns

**Figure 3:**
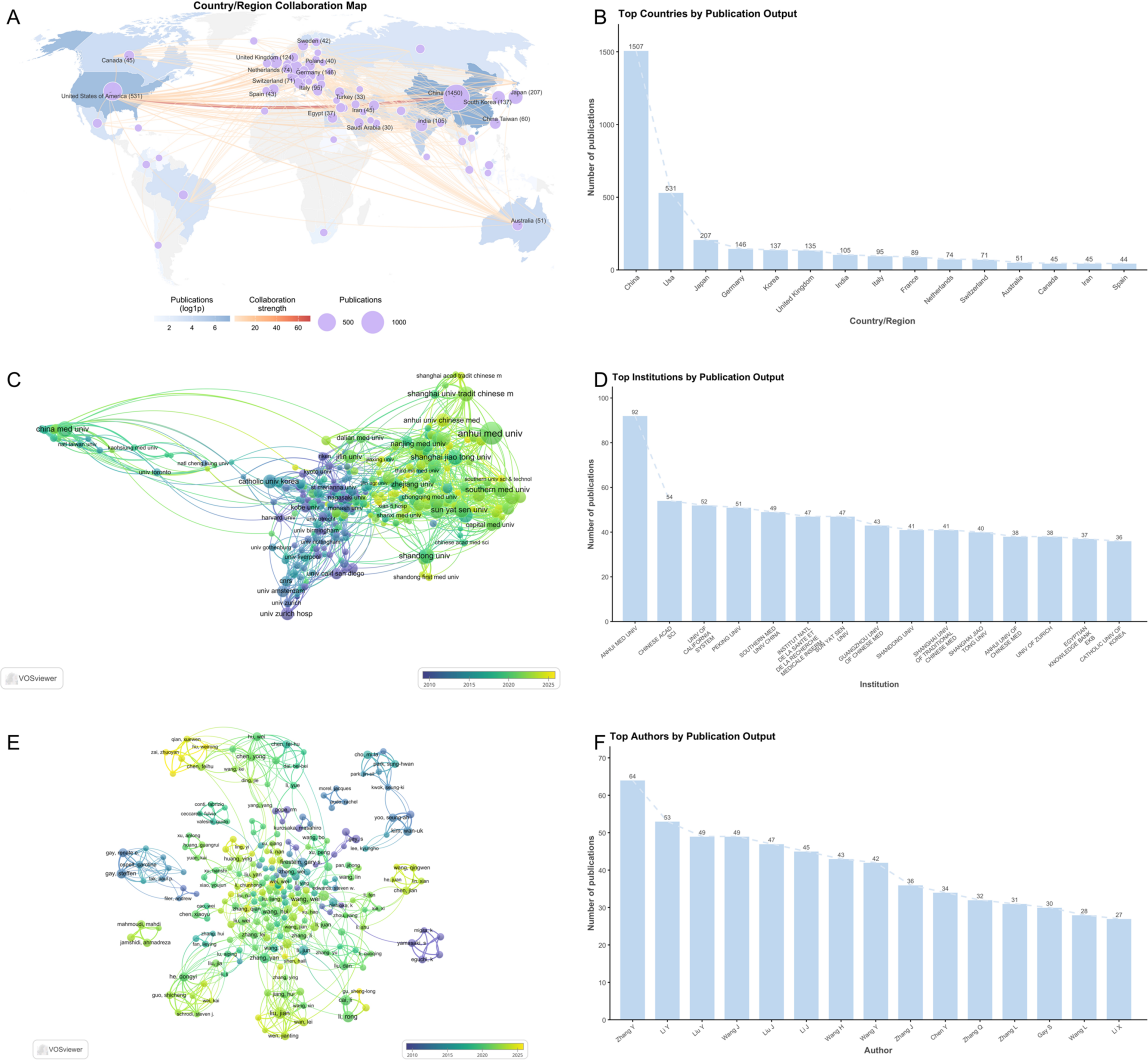
Global collaboration and major contributors. **(A)** World map of country-level publication output and international collaboration. **(B)** Top 15 countries/regions by publication output. **(C)** Institutional collaboration network generated by VOSviewer (minimum occurrence = 5; 332 items, 30 clusters, 1, 090 links, total link strength = 1, 661). **(D)** Top 15 institutions by publication output. **(E)** Author collaboration network generated by VOSviewer (minimum occurrence = 5; 264 items, 41 clusters, 645 links, total link strength = 1, 714). **(F)** Top 15 authors by publication output.

To further characterize the global research landscape, publication output and collaboration patterns at the country, institutional, and author levels were analyzed (**Figure 3**). At the country level, the world map showed broad international participation and cross-national collaboration (**Figure 3A**). The top 15 countries/regions by publication output are presented in **Figure 3B**. China ranked first with 1, 507 publications, followed by the United States (531), Japan (207), Germany (146), and South Korea (137), indicating that research output was concentrated in a limited number of leading countries/regions. To further distinguish publication volume from citation impact and international collaboration, country-level indicators were examined (**Supplementary Table 2**). China had the highest overall publication output, whereas the United States showed the highest total citations. Several countries with lower publication volume, including Germany, the United Kingdom, the Netherlands, Switzerland, and Australia, showed relatively high MCP ratios, indicating stronger international collaboration intensity. These findings suggest that publication output, citation impact, and international collaboration were not fully aligned across countries.

At the institutional level, the co-organization network generated by VOSviewer identified 332 items, 30 clusters, 1, 090 links, and a total link strength of 1, 661 (**Figure 3C**), showing a cluster-structured pattern of inter-institutional collaboration. The top 15 institutions by publication output are shown in **Figure 3D**. Anhui Medical University ranked first with 92 publications, followed by Chinese Academy of Sciences (54), University of California System (52), Peking University (51), and Southern Medical University China (49).

At the author level, the co-authorship network included 264 items, 41 clusters, 645 links, and a total link strength of 1, 714 (**Figure 3E**), suggesting the presence of multiple collaborative author groups. The top 15 authors by publication output are shown in **Figure 3F**. Zhang Y ranked first with 64 publications, followed by Li Y (53), Liu Y (49), Wang J (49), and Liu J (47).

Overall, the field was characterized by concentrated contributions from a limited number of highly productive countries, institutions, and authors, together with collaboration at multiple levels.

### 3.2 Knowledge base, thematic structure, and temporal dynamics

To characterize the knowledge base, thematic structure, and temporal dynamics of programmed cell death research in rheumatoid arthritis (RA), we performed integrated analyses based on reference co-citation, author keyword co-occurrence, and the temporal frequency characteristics of high-frequency keywords and major thematic domains. Reference co-citation analysis was used to identify the intellectual base of the field, author keyword analysis was used to map the thematic structure, and temporal analyses were used to summarize changes in major topics and thematic domains across the study period.

#### 3.2.1 Intellectual base revealed by reference co-citation analysis

**Figure 4:**
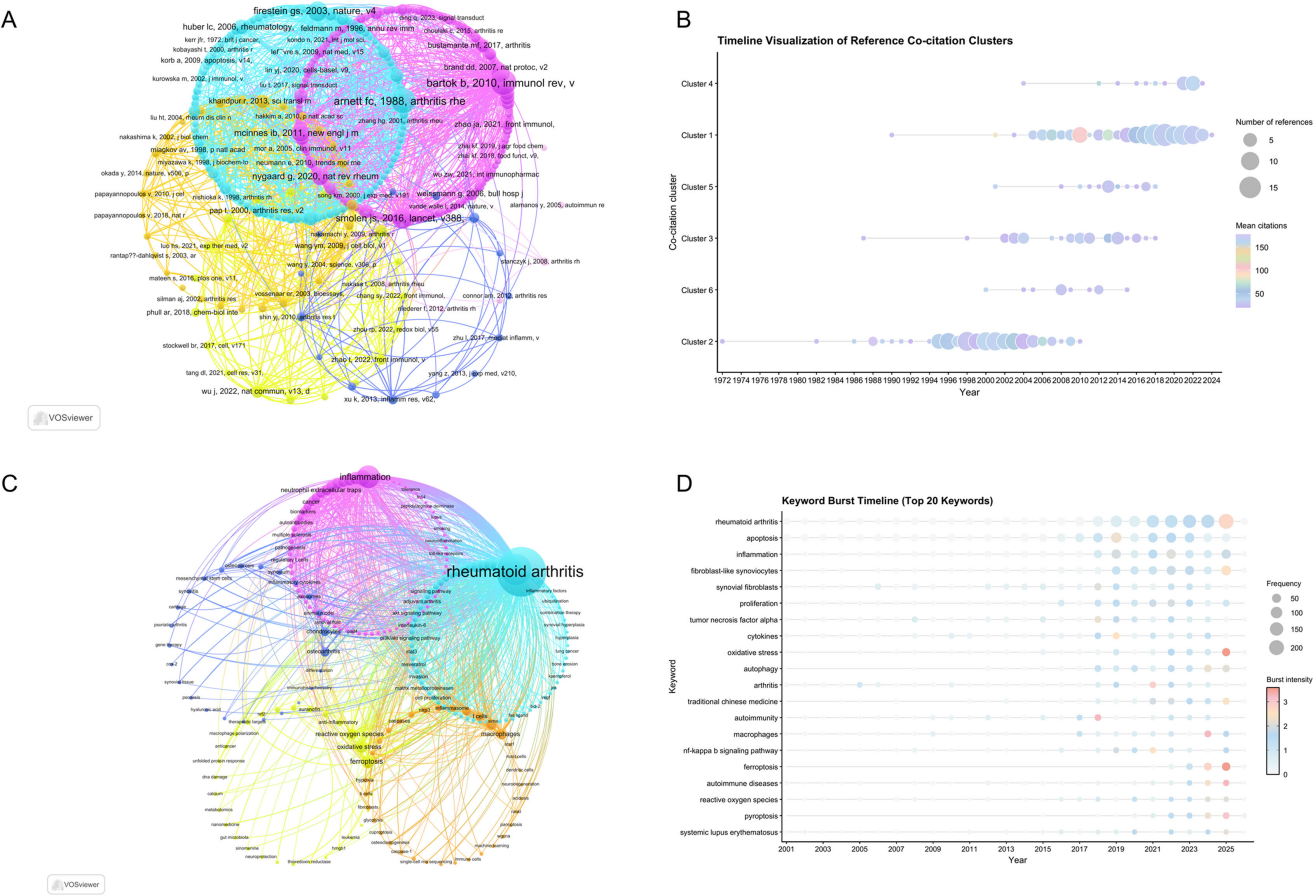
Intellectual base, thematic structure, and temporal dynamics. **(A)** Reference co-citation network generated using VOSviewer with a minimum citation threshold of 20. Each node represents a cited reference, and links indicate co-citation relationships. Node size reflects citation frequency, and colors denote distinct co-citation clusters. **(B)** Timeline bubble visualization of reference co-citation clusters. Each bubble represents the number of references within a given cluster-year combination, and bubble size is proportional to the number of references. Clusters are arranged along the temporal axis according to the publication years of their constituent references. **(C)** Author keyword co-occurrence network generated using VOSviewer with a minimum occurrence threshold of 5. Each node represents an author keyword, and links indicate co-occurrence relationships. Node size reflects keyword occurrence frequency, and colors denote thematic clusters. **(D)** Timeline of the top 20 high-frequency keywords. Bubble size represents annual keyword frequency, and color intensity reflects the relative prominence of each keyword over time based on year-wise standardized deviations from its average frequency.

Reference co-citation analysis identified six major clusters in the field (**Figures 4A, B**; **Table 1**). The two largest clusters formed the core intellectual base. Cluster 1 contained 123 references and showed the highest total citations and total link strength, indicating the strongest structural centrality in the co-citation network. The representative references in this cluster were mainly related to the general pathogenesis and conceptual framework of RA. Cluster 2 included 99 references and was characterized by an earlier mean publication year, with representative references focusing on classical RA mechanisms, synovial pathology, and inflammatory signaling.

**Table 1:** Main clusters in the reference co-citation network.

Cluster	No. of refs	Mean year	Total citations	TLS	Representative references	Putative theme
1	123	2016.3	4518	23456	Bartok B, 2010, *Immunol Rev*; Smolen JS, 2016, *Lancet*; Bottini N, 2013, *Nat Rev Rheumatol*	General pathogenesis and conceptual framework of rheumatoid arthritis
2	99	1999.3	3534	18012	Arnett FC, 1988, *Arthritis Rheum*; Firestein GS, 2003, *Nature*; Pope RM, 2002, *Nat Rev Immunol*	Classical rheumatoid arthritis mechanisms, synovial pathology, and inflammatory signaling
3	31	2008.7	805	4378	Khandpur R, 2013, *Sci Transl Med*; Brinkmann V, 2004, *Science*; Wang YM, 2009, *J Cell Biol*	Neutrophil extracellular traps, neutrophil-mediated immunity, and autoimmunity
4	19	2018.9	563	4549	Wu J, 2022, *Nat Commun*; Dixon SJ, 2012, *Cell*; Zhao T, 2022, *Front Immunol*	Ferroptosis, oxidative stress, and newly emerging cell death pathways
5	15	2012.7	417	3388	Kato M, 2014, *Arthritis Rheumatol*; Xu K, 2013, *Inflamm Res*; Shin YJ, 2010, *Arthritis Res Ther*	Cell-death-related inflammatory mechanisms in joint damage
6	11	2008.4	282	1562	Kinne RW, 2000, *Arthritis Res*; Stanczyk J, 2008, *Arthritis Rheum*; Choy E, 2012, *Rheumatology*	Fibroblast regulation, microRNA-related mechanisms, and disease modulation

Clusters were identified from the reference co-citation network generated using VOSviewer. “No. of refs” indicates the number of references included in each cluster. “Mean year, “ “total citations, “ and “TLS” (total link strength) were derived from node-level attributes of the co-citation network. Representative references were selected according to citation frequency and total link strength within each cluster. Putative thematic labels were assigned based on the content of the leading references and are intended to summarize the major knowledge domains represented by each cluster.

The remaining clusters showed more specialized knowledge domains. Cluster 3 was mainly associated with neutrophil extracellular traps, neutrophil-mediated immunity, and autoimmunity. Cluster 4 had the most recent mean publication year and was characterized by ferroptosis, oxidative stress, and newly emerging forms of regulated cell death. Further inspection of cross-cluster links showed that Cluster 4 was structurally connected mainly with Cluster 1 and, to a lesser extent, Cluster 2. The total link strength between Cluster 4 and Cluster 1 was 2, 205, whereas that between Cluster 4 and Cluster 2 was 202. Several Cluster 4 references showed relatively strong links with Clusters 1–2, including Wu et al. (19), Zhao et al. (20), Dixon et al. (21), Phull et al. (22), and Xie et al. (23) (**Supplementary Table 3**). Clusters 5 and 6 were linked to cell-death-related inflammatory mechanisms, fibroblast regulation, and microRNA-related disease modulation. Together, these clusters outlined the main intellectual components of the field from classical RA pathology to more recent regulated cell death research.

The timeline bubble visualization further showed the temporal distribution of references across clusters (**Figure 4B**). Earlier clusters were mainly distributed in foundational studies on RA classification, synovial inflammation, and immune-mediated joint damage, whereas later clusters included references related to ferroptosis and oxidative-stress-associated cell death. Bubble size indicated the number of references within each cluster-year combination, showing that the intellectual structure of the field gradually extended from classical RA mechanisms to more recently emphasized regulated cell death topics. The top co-cited references were dominated by landmark articles and reviews on RA pathogenesis and inflammatory regulation, together with more recent references related to specific cell death pathways (**Table 2**).

**Table 2:** Top co-cited references.

Rank	Cited reference	Year	Co-citation counts	Total link strength	Cluster
1	Bartok B, *Immunol Rev*	2010	320	1982	1
2	Arnett FC, *Arthritis Rheum*	1988	312	1272	2
3	Smolen JS, *Lancet*	2016	282	1280	1
4	Firestein GS, *Nature*	2003	264	1048	2
5	Bottini N, *Nat Rev Rheumatol*	2013	261	1094	1
6	McInnes IB, *N Engl J Med*	2011	251	956	1
7	Nygaard G, *Nat Rev Rheumatol*	2020	191	1090	1
8	Pope RM, *Nat Rev Immunol*	2002	178	854	2
9	Aletaha D, *JAMA*	2018	110	729	1
10	Huber LC, *Rheumatology*	2006	102	653	2
11	Smolen JS, *Nat Rev Dis Primers*	2018	100	679	1
12	Aletaha D, *Arthritis Rheum*	2010	100	450	1
13	McInnes IB, *Lancet*	2017	97	641	1
14	Scott DL, *Lancet*	2010	92	564	1
15	Baier A, *Curr Opin Rheumatol*	2003	91	604	2

Top co-cited references were identified from the reference co-citation network generated using VOSviewer and ranked by co-citation counts. Co-citation counts indicate the frequency with which a reference was cited together with other references across the retrieved literature, whereas total link strength reflects the overall strength of its co-citation relationships within the network. Obvious malformed entries were excluded during manual verification, and cluster numbers correspond to the co-citation clusters shown in **Figures 4A, B**.

#### 3.2.2 Thematic structure identified from author keywords

Author keyword co-occurrence analysis identified five major thematic clusters in the field (**Figure 4C**; **Table 3**). Cluster 2 was the largest cluster and showed the highest total occurrences and total link strength. It was centered on terms such as rheumatoid arthritis, apoptosis, fibroblast-like synoviocytes, NF-kappa B signaling pathway, and traditional Chinese medicine, indicating that synovial fibroblast activation, apoptotic regulation, and classical inflammatory signaling constituted a major thematic component of the field. Cluster 1 included inflammation, autophagy, autoimmunity, autoimmune diseases, cytokines, neutrophils, and neutrophil extracellular traps, reflecting a broad theme related to inflammatory and autoimmune regulation.

**Table 3:** Main thematic clusters identified from author keywords.

Cluster	No.	Mean year	Freq.	TLS	Representative keywords	Putative theme
1	77	2017.8	1599	4077	inflammation; autophagy; autoimmunity; autoimmune diseases; arthritis	Inflammation, autophagy, and autoimmune regulation
2	75	2017.8	3380	7772	rheumatoid arthritis; apoptosis; fibroblast-like synoviocytes; nf-kappa b signaling pathway; traditional chinese medicine	Rheumatoid arthritis, apoptosis, and synovial fibroblast signaling
3	35	2019.5	478	1175	macrophages; pyroptosis; t cells; osteoclasts; inflammasome	Pyroptosis, inflammasome activation, and immune-cell-mediated bone destruction
4	30	2020.5	481	1077	ferroptosis; oxidative stress; reactive oxygen species; anti-inflammatory; cell death	Ferroptosis, oxidative stress, and redox-related therapeutic targeting
5	25	2016.8	341	849	osteoarthritis; chondrocytes; animal model; exosomes; inflammatory cytokines	Cartilage biology, extracellular vesicles, and related joint disease models

Thematic clusters were identified from the author keyword co-occurrence network generated using VOSviewer. “No.” indicates the number of keywords included in each cluster. “Mean year, “ “Freq.”, and “TLS” (total link strength) were derived from node-level attributes of the keyword network. Representative keywords were selected according to occurrence frequency and total link strength within each cluster. Putative thematic labels were assigned based on the semantic content of the leading keywords and are intended to summarize the major thematic domains represented by each cluster.

Clusters 3 and 4 were more strongly associated with emerging mechanistic directions. Cluster 3 contained macrophages, pyroptosis, T cells, osteoclasts, inflammasome, NLRP3, caspase-1, and necroptosis, and also included method-related terms such as bioinformatics, machine learning, single-cell RNA sequencing, and WGCNA. Cluster 4 included ferroptosis, oxidative stress, reactive oxygen species, Nrf2, antioxidants, and anti-inflammatory, together with terms related to therapeutic exploration such as nanomedicine, targeted therapy, and gut microbiota. Cluster 5 comprised osteoarthritis, chondrocytes, animal model, exosomes, and inflammatory cytokines, corresponding to an additional theme associated with cartilage biology, extracellular vesicle-mediated regulation, and related joint disease models.

Based on the composition of these clusters, the thematic structure of the field could be summarized into five major mechanistic domains: inflammatory and autoimmune regulation, synovial fibroblast dysfunction and classical signaling pathways, inflammasome-related and emerging programmed cell death pathways, oxidative stress-related cell death and redox regulation, and cartilage biology, extracellular vesicle-mediated regulation, and related joint injury/repair. Among these, the first two domains represented the most structurally central components of the keyword network, whereas the latter domains reflected more specialized and mechanistically focused research directions.

Overall, the keyword co-occurrence network showed that programmed cell death research in RA was organized around interconnected themes involving inflammatory immunity, abnormal activation of synovial stromal cells, regulated cell death mechanisms, oxidative stress-related processes, and joint tissue damage and repair.

#### 3.2.3 Temporal characteristics of high-frequency keywords and major thematic domains

The temporal analysis of high-frequency keywords showed marked differences in total frequency, peak annual frequency, prominence intensity, and temporal span among the top 20 terms (**Figure 4D**; **Table 4**). Rheumatoid arthritis, apoptosis, inflammation, and arthritis had the highest total frequencies, indicating that these were persistent core topics in the field. Fibroblast-like synoviocytes, synovial fibroblasts, and proliferation also maintained relatively broad temporal spans, corresponding to the sustained prominence of synovial stromal-cell-related research.

**Table 4:** Temporal frequency characteristics of the top 20 keywords.

Rank	Keyword	Total freq.	Peak freq.	Max prominence	Onset	End	Span, y
1	oxidative stress	195	28	3.074	2015	2025	11
2	ferroptosis	100	33	3.040	2022	2025	4
3	autoimmune diseases	83	11	2.550	2012	2025	14
4	autoimmunity	127	16	2.469	2007	2024	18
5	macrophages	126	15	2.430	2009	2025	17
6	pyroptosis	70	18	3.065	2020	2025	7
7	arthritis	148	18	2.287	2005	2024	20
8	rheumatoid arthritis	1405	183	2.733	2017	2025	9
9	cytokines	218	19	2.083	2008	2024	17
10	fibroblast-like synoviocytes	447	47	2.601	2016	2025	10
11	nf-kappa b signaling pathway	104	13	2.532	2008	2025	18
12	traditional chinese medicine	127	18	2.380	2013	2025	13
13	apoptosis	925	97	2.312	2014	2025	12
14	autophagy	185	25	2.442	2018	2025	8
15	tumor necrosis factor alpha	232	22	2.076	2005	2024	20
16	systemic lupus erythematosus	70	10	2.000	2002	2025	24
17	reactive oxygen species	71	10	2.364	2008	2025	18
18	synovial fibroblasts	268	24	1.957	2006	2024	19
19	inflammation	543	69	2.656	2018	2025	8
20	proliferation	261	26	2.113	2016	2025	10

Temporal characteristics were calculated from yearly keyword frequencies in the retrieved literature. “Total freq.” indicates the total frequency of each keyword across the study period, and “Peak freq.” indicates its highest annual frequency. “Max prominence” represents the highest year-specific prominence value of each keyword, derived from standardized deviations of annual frequency relative to its own average level across the study period. “Onset” and “End” indicate the first and last years in which each keyword showed positive prominence intensity in the dataset, and “Span, y” denotes the corresponding duration in years. This table complements the keyword timeline visualization by summarizing the temporal distribution and relative prominence of the top 20 keywords.

Several keywords showed more recent temporal concentration and relatively high prominence intensity. Ferroptosis and pyroptosis were characterized by later onset and short prominence spans, but both showed high prominence intensity in recent years. Oxidative stress and reactive oxygen species also displayed notable temporal prominence, while macrophages remained active across a broader time range. By contrast, some treatment-related and pathway-related terms showed more limited temporal prominence. Traditional Chinese medicine showed a relatively recent prominence range, whereas NF-kappa B signaling pathway and tumor necrosis factor alpha showed comparatively lower prominence intensity within the selected top-20 keyword set.

**Figure 5:**
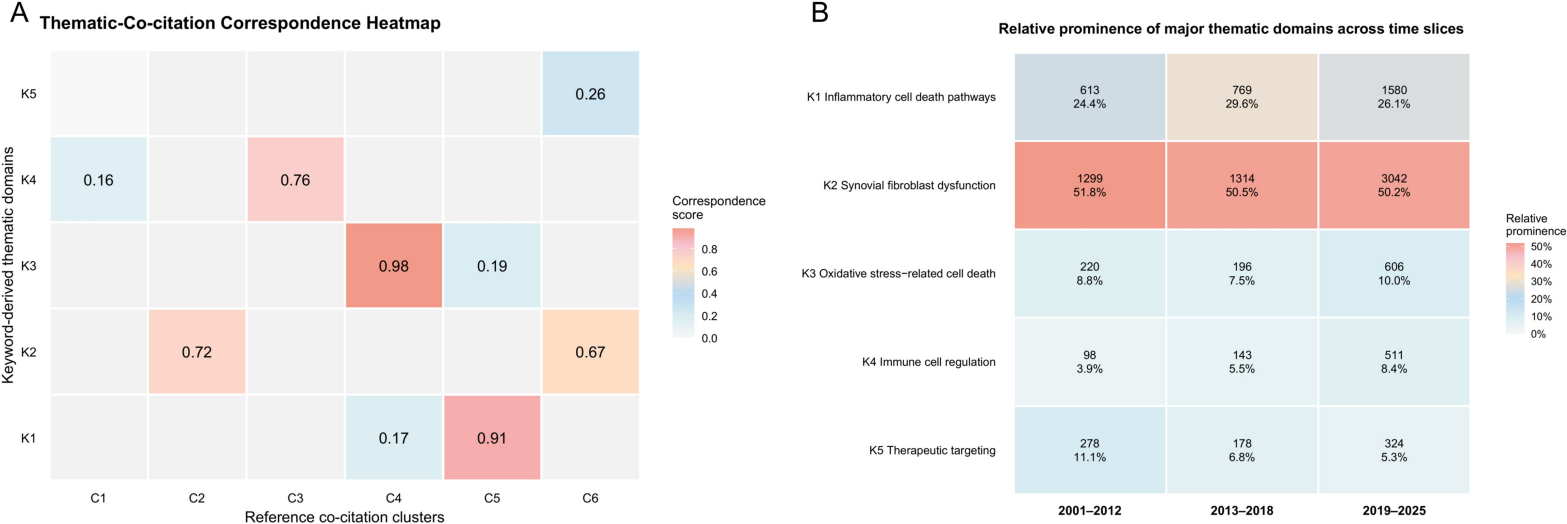
Structural correspondence and temporal prominence of keyword-derived thematic domains. **(A)** Correspondence heatmap between keyword-derived thematic domains and reference co-citation clusters. Rows represent five keyword-derived thematic domains (K1–K5), and columns represent six reference co-citation clusters (C1–C6). Cell values are cosine-similarity correspondence scores from weighted term vectors; higher scores indicate stronger structural correspondence. The top two correspondence scores are highlighted. K1, inflammatory cell death pathways; K2, synovial fibroblast dysfunction; K3, oxidative stress-related cell death; K4, immune cell regulation; K5, therapeutic targeting. C1–C6 correspond to the six reference co-citation clusters shown in **Table 1**. **(B)** Heatmap of the relative prominence of major thematic domains across three time slices (2001–2012, 2013–2018, and 2019–2025). Cell color indicates the relative prominence of each thematic domain within a given period, and the values shown in the cells represent the corresponding frequency and relative proportion.

At the domain level, the five keyword-derived thematic domains were coded as K1–K5 in **Figure 5** and **Table 5**, corresponding to inflammatory cell death pathways (K1), synovial fibroblast dysfunction (K2), oxidative stress-related cell death (K3), immune cell regulation (K4), and therapeutic targeting (K5). The six reference co-citation clusters were coded as C1–C6, corresponding to the six clusters shown in **Figure 5A** and **Table 1**. The relative prominence heatmap and the corresponding summary data showed variation in the distribution of these five thematic domains across the three time slices (**Figure 5B**; **Table 5**). K2 remained the dominant domain throughout the study period, accounting for 51.8% of all domain-assigned frequencies in 2001–2012, 50.5% in 2013–2018, and 50.2% in 2019–2025. K1 also showed sustained prominence across all three periods, with relative proportions of 24.4%, 29.6%, and 26.1%, respectively. By contrast, K4 increased from 3.9% in the earliest period to 8.4% in 2019–2025, indicating a gradual rise in the relative prominence of immune-cell-related themes. K3 showed a lower proportion in the earlier two periods but increased to 10.0% in the most recent period, consistent with the recent expansion of oxidative-stress- and ferroptosis-related research. In contrast, K5 showed a declining relative proportion over time, from 11.1% to 5.3%, indicating that this domain became relatively less prominent as the field expanded toward more specific mechanistic themes.

**Table 5:** Structural correspondence and temporal prominence of keyword-derived thematic domains.

Domain	Top cluster	Top score	Second cluster	Second score	2001–2012, n (%)	2013–2018, n (%)	2019–2025, n (%)
K1	C5	0.906	C4	0.175	613 (24.4)	769 (29.6)	1580 (26.1)
K2	C2	0.718	C6	0.671	1299 (51.8)	1314 (50.5)	3042 (50.2)
K3	C4	0.980	C5	0.187	220 (8.8)	196 (7.5)	606 (10.0)
K4	C3	0.758	C1	0.155	98 (3.9)	143 (5.5)	511 (8.4)
K5	C6	0.255	C1	0.000	278 (11.1)	178 (6.8)	324 (5.3)

This table summarizes the structural correspondence between the five keyword-derived thematic domains and the six reference co-citation clusters, together with the temporal prominence of each thematic domain across the three predefined time slices (2001–2012, 2013–2018, and 2019–2025). “Top cluster” and “Second cluster” indicate the two reference co-citation clusters with the highest correspondence to each thematic domain, and “Top score” and “Second score” represent the corresponding cosine similarity values. Temporal prominence values are presented as frequency and relative proportion within each time slice. K1, inflammatory cell death pathways; K2, synovial fibroblast dysfunction; K3, oxidative stress-related cell death; K4, immune cell regulation; K5, therapeutic targeting. C1–C6 correspond to the six reference co-citation clusters shown in **Figure 5A** and **Table 1**.

The correspondence heatmap further showed that the five keyword-derived thematic domains were not evenly distributed across the six reference co-citation clusters (**Figure 5A**; **Table 5**). Higher correspondence scores were concentrated in a limited number of domain-cluster pairs, whereas most remaining cells showed relatively lower values. The strongest correspondence was observed between K1 and C5 (0.906), K3 and C4 (0.980), and K4 and C3 (0.758). K2 showed relatively high correspondence with both C2 (0.718) and C6 (0.671), whereas K5 showed its highest correspondence with C6 (0.255), with much weaker correspondence values overall than the other thematic domains. These patterns indicate that the five thematic domains were differentially anchored in specific segments of the reference co-citation structure.

Overall, the temporal and structural analyses showed that persistent core topics coexisted with more recently intensified mechanistic domains, while the thematic domains identified from author keywords also showed differentiated temporal prominence and structural correspondence across the study period.

#### 3.2.4 Sensitivity analyses

Sensitivity analyses were conducted to examine the robustness of the main network findings (**Supplementary Table 1**). For author keyword co-occurrence analysis, changing the minimum occurrence threshold from the main value of 5 to 4 or 6 yielded 326 and 195 items, respectively, while the number of clusters remained 5 across all settings. The main thematic domains were retained, indicating that the keyword-based thematic structure was stable.

For reference co-citation analysis, changing the minimum citation threshold from the main value of 20 to 18 or 22 yielded 354 and 238 references, respectively. The core intellectual domains remained broadly consistent, although smaller or more recent clusters showed greater threshold sensitivity. Dataset-source sensitivity analysis showed that excluding the seven PubMed-only records did not change the network-level results of either author keyword co-occurrence or reference co-citation analysis. These findings indicate that the main network-level thematic and intellectual structures were robust to the tested parameter and dataset-source changes.

### 3.3 Source landscape and disciplinary diffusion

#### 3.3.1 Core source journals and their knowledge relationships

**Figure 6:**
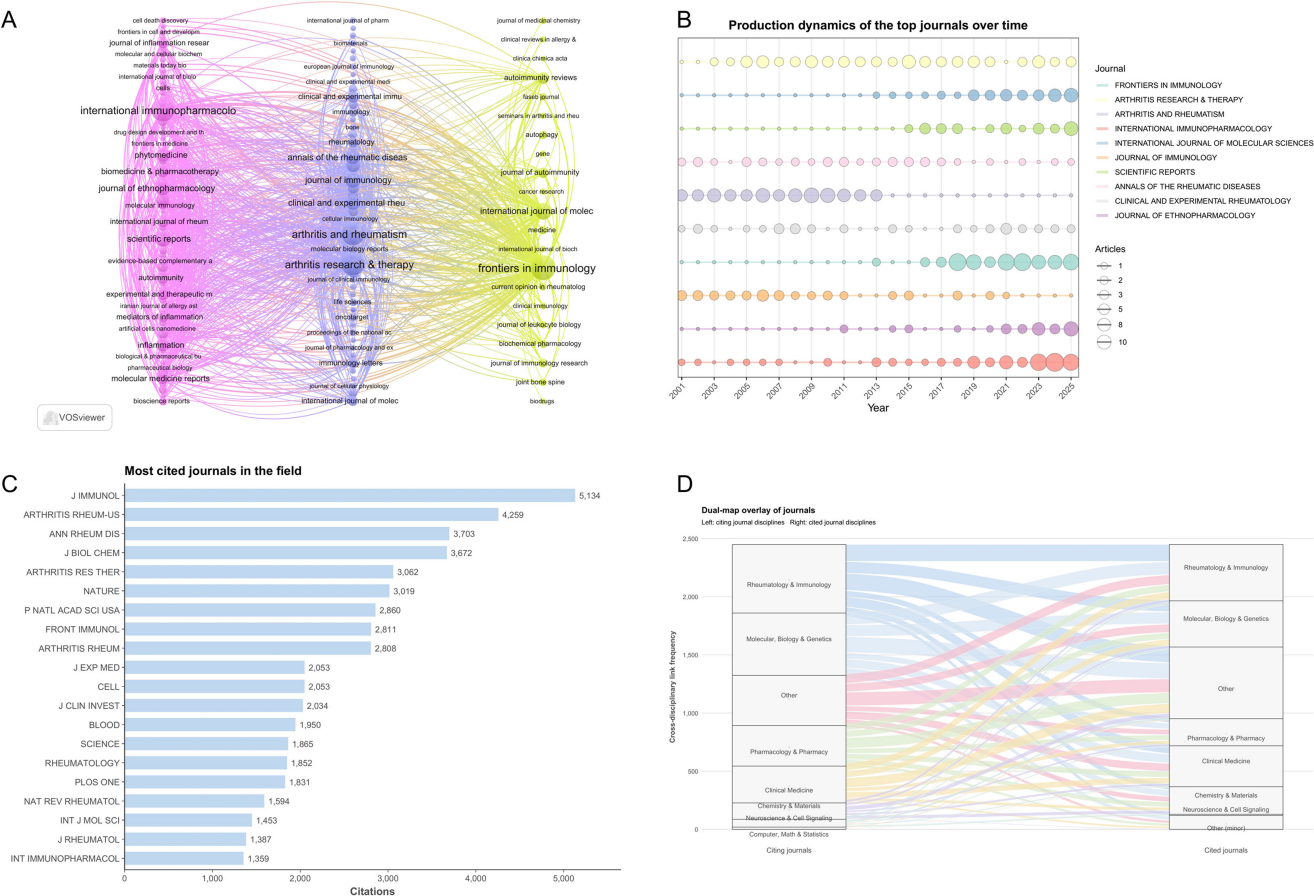
Source landscape and disciplinary diffusion. **(A)** Journal bibliographic coupling network generated by VOSviewer at the source level with a minimum threshold of 5 documents per source. Of 832 sources, 141 met the threshold and were included in the network, yielding 3 clusters, 9, 069 links, and a total link strength of 294, 579. Each node represents a journal, node size reflects total link strength, links indicate bibliographic coupling relationships, and colors denote journal clusters. **(B)** Production dynamics of the top journals over time. Bubble size represents the annual number of publications, and colored trajectories indicate changes in journal productivity across the study period. **(C)** Most cited journals in the field. The bar chart presents the journals most frequently cited by the retrieved publications. **(D)** Dual-map overlay of journals showing the major citation flows between citing and cited journal domains. The left side represents the disciplinary distribution of citing journals, and the right side represents that of cited journals. Curved paths indicate citation flows between disciplinary domains.

Source-level analysis revealed a relatively concentrated journal landscape in programmed cell death research in rheumatoid arthritis (RA). Bibliographic coupling analysis of journals showed that, with the minimum threshold set at 5 documents per source, 141 of 832 sources met the inclusion criteria and formed a network containing 3 clusters, 9, 069 links, and a total link strength of 294, 579 (**Figure 6A**). The network displayed a closely connected structure, with major journals grouped into three source clusters according to similarities in cited-reference patterns.

The most active journals in the field are presented in **Table 6**. Frontiers in Immunology ranked first in publication output (109 publications), followed by Arthritis Research & Therapy (102) and Arthritis and Rheumatism (101). In terms of bibliographic coupling strength, Frontiers in Immunology also had the highest total link strength (40, 299), followed by Arthritis Research & Therapy (30, 200) and Arthritis and Rheumatism (27, 269), indicating their central positions in the source network. Other active journals included International Immunopharmacology, International Journal of Molecular Sciences, Journal of Immunology, Scientific Reports, Annals of the Rheumatic Diseases, Clinical and Experimental Rheumatology, and Journal of Ethnopharmacology.

**Table 6:** Top active journals.

Rank	Journal	Pubs	TLS	Citations	Cluster
1	Frontiers in Immunology	109	40299	6477	3
2	Arthritis Research & Therapy	102	30200	4865	2
3	Arthritis and Rheumatism	101	27269	7483	2
4	International Immunopharmacology	93	26092	2053	1
5	International Journal of Molecular Sciences	49	15256	1053	3
6	Journal of Immunology	47	11027	3962	2
7	Scientific Reports	41	11728	819	1
8	Annals of the Rheumatic Diseases	40	11654	3414	2
9	Clinical and Experimental Rheumatology	40	10695	1079	2
10	Journal of Ethnopharmacology	37	9718	630	1

This table presents the leading source journals in programmed cell death research in rheumatoid arthritis (RA), ranked by publication output. “Pubs” indicates the number of retrieved articles published in each journal. “TLS” (total link strength) refers to the overall strength of bibliographic coupling links between a given journal and other journals in the source network. “Citations” indicates the total citations received by publications from each journal within the dataset. “Cluster” denotes the journal cluster identified in the bibliographic coupling network.

The temporal production dynamics of the leading journals are shown in **Figure 6B**. Several journals maintained sustained output across the study period, whereas others showed more evident growth in recent years. Together, these patterns indicate that the source landscape of this field consists of a limited number of highly productive journals accompanied by additional journals with more recent or topic-specific contributions.

The most cited journals in the field are shown in **Figure 6C**. Journal of Immunology ranked first by citation frequency (5, 134 citations), followed by Arthritis & Rheumatism (4, 259), Annals of the Rheumatic Diseases (3, 703), Journal of Biological Chemistry (3, 672), and Arthritis Research & Therapy (3, 062). These highly cited journals represent the principal cited sources underpinning the knowledge base of programmed cell death research in RA.

#### 3.3.2 Disciplinary diffusion revealed by the dual-map overlay

The dual-map overlay revealed the main disciplinary citation flows between citing and cited journal domains (**Figure 6D**; **Table 7**). The dominant citation path was from Rheumatology & Immunology to Rheumatology & Immunology (link frequency = 20, 419), accounting for 11.0% of all links and 32.8% of links within the citing domain. Another major path was from Rheumatology & Immunology to Other (19, 995; 10.8% of total links), followed by Molecular, Biology & Genetics to Other (17, 285; 9.3%) and Other to Other (13, 290; 7.2%).

**Table 7:** Main citation paths identified from the dual-map overlay.

Rank	Citation path	Citing domain	Cited domain	Links	Total share, %	Domain share, %
1	Rheumatology & Immunology → Rheumatology & Immunology	Rheumatology & Immunology	Rheumatology & Immunology	20419	11.0%	32.8%
2	Rheumatology & Immunology → Other	Rheumatology & Immunology	Other	19995	10.8%	32.1%
3	Molecular, Biology & Genetics → Other	Molecular, Biology & Genetics	Other	17285	9.3%	35.2%
4	Other → Other	Other	Other	13290	7.2%	41.5%
5	Molecular, Biology & Genetics → Rheumatology & Immunology	Molecular, Biology & Genetics	Rheumatology & Immunology	11210	6.1%	22.8%
6	Molecular, Biology & Genetics → Molecular, Biology & Genetics	Molecular, Biology & Genetics	Molecular, Biology & Genetics	9403	5.1%	19.1%
7	Rheumatology & Immunology → Molecular, Biology & Genetics	Rheumatology & Immunology	Molecular, Biology & Genetics	9301	5.0%	14.9%
8	Pharmacology & Pharmacy → Other	Pharmacology & Pharmacy	Other	7902	4.3%	39.5%
9	Rheumatology & Immunology → Clinical Medicine	Rheumatology & Immunology	Clinical Medicine	7396	4.0%	11.9%
10	Clinical Medicine → Other	Clinical Medicine	Other	6474	3.5%	37.5%

This table summarizes the principal citation paths identified from the dual-map overlay of journals. “Citation path” represents the directional flow from the disciplinary domain of citing journals to that of cited journals. “Links” indicates the number of citation links observed for each path. “Total share, %” refers to the proportion of a given path among all citation links in the overlay, and “Domain share, %” indicates the relative contribution of a given path within the corresponding citing discipline.

Additional major paths connected Molecular, Biology & Genetics with both Rheumatology & Immunology (11, 210; 6.1%) and Molecular, Biology & Genetics itself (9, 403; 5.1%). Citation flows from Rheumatology & Immunology to Molecular, Biology & Genetics (9, 301; 5.0%) and to Clinical Medicine (7, 396; 4.0%) were also prominent. In addition, Pharmacology & Pharmacy to Other (7, 902; 4.3%) and Clinical Medicine to Other (6, 474; 3.5%) constituted additional major citation paths. Overall, the dual-map overlay showed that knowledge diffusion in this field was primarily concentrated among rheumatology/immunology, molecular-biological, and clinical source domains.

## 4 Discussion

### 4.1 General overview

The present study provides a comprehensive bibliometric overview of global research on programmed cell death (PCD) in rheumatoid arthritis (RA) from 2001 to 2025. Overall, the findings show that this field has expanded steadily, with a clear increase in publication output during the later years of the study period, reflecting growing interest in the role of regulated cell death in RA pathogenesis. This recent increase should be interpreted with some caution because publication indexing and citation accumulation may be incomplete for the terminal year. More importantly, the knowledge structure and thematic patterns indicate that research on PCD in RA has evolved from an earlier framework centered mainly on apoptosis and synovial pathology toward a broader and increasingly differentiated mechanistic landscape involving pyroptosis, ferroptosis, inflammasome activation, oxidative stress-related injury, and tissue microenvironment regulation (24–26). This shift suggests that PCD is no longer viewed simply as a downstream consequence of cellular damage, but increasingly as an active mechanistic framework for understanding how inflammatory signaling, immune-cell activation, stromal dysfunction, and joint tissue injury are interconnected in RA (10, 27). In this sense, PCD has become an important organizing concept in the field, linking classical disease mechanisms with newly emerging directions in inflammatory and cell-death biology (28, 29). Taken together, these findings suggest that PCD research in RA is moving from a relatively narrow, pathology-oriented perspective toward a more integrated view that connects molecular cell death programs with the broader pathophysiological architecture of RA (30).

### 4.2 Developmental trajectory, intellectual foundation, and thematic evolution

A major finding of this study is the clear and sustained upward trajectory of the field, particularly the marked increase in publication output during the later years of the study period. This pattern suggests that PCD in RA has shifted from a relatively focused mechanistic topic to a more visible and active research domain. In the earlier stage, the field was mainly embedded in the classical pathological framework of RA, especially synovial inflammation, immune dysregulation, and the abnormal survival of fibroblast-like synoviocytes (FLS) (31–33). Within this framework, apoptosis occupied a central position because defective apoptotic regulation offered a compelling explanation for synovial hyperplasia, pannus formation, and persistent inflammatory tissue expansion (8, 34). As the field developed, however, the concept of PCD in RA broadened substantially. Cell death was no longer viewed only from the perspective of insufficient apoptotic clearance, but increasingly as a mechanistic hub linking inflammation, immune activation, oxidative stress, and joint tissue injury (9, 12, 35). The rapid rise in publication output in recent years was a prominent feature of the present bibliometric dataset. This pattern may reflect not only growing attention to RA pathogenesis itself, but also the broader expansion of regulated cell death research across immunology, inflammatory disease biology, and translational medicine.

Although the growth pattern demonstrates increasing academic attention, the co-citation analysis shows that the field remains intellectually anchored in the classical literature on RA. The largest and most structurally central co-citation clusters were dominated by landmark publications on general RA pathogenesis, synovial pathology, inflammatory signaling, and immune-mediated joint destruction (1, 36). This finding is important because it indicates that research on PCD in RA has not developed as an isolated mechanistic niche. Rather, it has emerged through reinterpretation and extension of established RA pathobiology (37). In other words, the broader RA literature provides the conceptual disease framework, whereas PCD-related studies refine this framework by focusing on specific forms of cellular injury, regulated death signaling, and inflammation-associated tissue responses (38–40). This cumulative pattern suggests that the development of the field has been integrative rather than disruptive. The classical understanding of RA remains the intellectual foundation, and newer PCD-related concepts have been progressively layered onto that foundation.

At the same time, the co-citation clusters also reveal a clear process of specialization within the field. In addition to the large foundational clusters related to general RA mechanisms, more recent and relatively focused clusters emerged around ferroptosis, oxidative stress, neutrophil extracellular traps, and cell-death-related inflammatory injury (41–43). This structural pattern suggests that the field is undergoing a transition from a broad disease-oriented framework to a more differentiated mechanistic architecture. The cross-cluster linkage analysis further showed that the recent ferroptosis/oxidative-stress cluster was connected mainly with the classical RA pathogenesis cluster and, to a lesser extent, the synovial pathology and inflammatory signaling cluster. This pattern indicates a structural association between redox-regulated cell death and the established RA knowledge base, rather than a fully isolated emerging topic. This interpretation is consistent with the foundational definition of ferroptosis as an iron-dependent form of regulated cell death and with experimental evidence linking ferroptotic cell death to synovial fibroblast regulation in arthritis models (19, 21). Such differentiation is highly meaningful in the context of RA, because the disease is not driven by a single pathogenic axis but instead involves the coordinated action of stromal cells, immune cells, cytokine networks, redox imbalance, and tissue-destructive pathways (44–46). The emergence of these specialized co-citation clusters indicates that PCD is increasingly being studied not simply as an endpoint of cellular damage, but as an active contributor to inflammatory amplification, immune-cell crosstalk, and pathological remodeling of the synovial microenvironment (47, 48).

Among the results of the present study, the keyword co-occurrence analysis and its related temporal and structural extensions provide the most direct insight into how the thematic core of the field has evolved. Five major thematic clusters were identified, spanning inflammatory and autoimmune regulation, synovial fibroblast dysfunction and apoptosis-related signaling, inflammasome-related and emerging PCD pathways, oxidative stress-associated cell death and redox regulation, and cartilage biology or extracellular-vesicle-related joint disease models. These clusters indicate that current research in this area is no longer organized around a single dominant mechanism. Instead, it is structured as a multi-dimensional system in which different forms of regulated cell death intersect with specific cellular compartments and pathological processes in RA (9).

Among these domains, the continued centrality of apoptosis, FLS-related terms, and inflammatory signaling pathways indicates that classical stromal pathology still occupies a foundational position in the field. This persistence is biologically plausible. FLS dysfunction remains one of the most characteristic pathological features of RA, and resistance to apoptosis continues to offer a strong explanatory framework for synovial hyperplasia, invasive pannus behavior, and chronic local inflammation (49–51). The long-term prominence of terms such as apoptosis, fibroblast-like synoviocytes, synovial fibroblasts, inflammation, and rheumatoid arthritis suggests that the field has maintained a stable core around which newer themes have developed. The appearance of traditional Chinese medicine within the keyword cluster centered on RA, apoptosis, fibroblast-like synoviocytes, and inflammatory signaling also deserves attention. This pattern suggests that at least part of the TCM-related literature in this field is framed using mainstream mechanistic vocabulary, particularly apoptosis, synovial fibroblast regulation, inflammatory signaling, oxidative stress, and regulated cell death (5, 6, 43).

However, the thematic structure also makes it clear that recent research has expanded beyond this classical framework. In particular, the cluster involving macrophages, pyroptosis, inflammasome, NLRP3, caspase-1, and necroptosis reflects increasing attention to inflammatory forms of cell death that directly connect innate immune activation with tissue injury (52, 53). This shift is especially significant because it broadens the interpretive scope of RA pathogenesis. Pyroptosis is not merely another death pathway added to the existing literature; it introduces a mechanistic model in which cell death itself actively drives cytokine release, amplifies inflammatory signaling, and contributes to the persistence of synovitis (10, 35). In this context, the increased prominence of pyroptosis-related terms likely reflects the field’s growing interest in how inflammatory cell death bridges molecular signaling and disease-level pathology.

A similar pattern is evident for ferroptosis and oxidative stress-related themes. The keyword cluster centered on ferroptosis, reactive oxygen species, antioxidants, Nrf2, and anti-inflammatory strategies, together with the corresponding co-citation cluster showing the most recent mean publication year, suggests that redox-regulated cell death has become one of the most rapidly emerging directions in the field. This trend is noteworthy because ferroptosis provides a mechanistic framework that links oxidative stress, iron metabolism, lipid peroxidation, and tissue injury—processes that are all highly relevant to chronic inflammatory joint damage (30, 54). Compared with apoptosis, which is more deeply embedded in the classical explanation of synovial proliferation and survival imbalance, ferroptosis reflects a more recent conceptual move toward metabolic stress, redox imbalance, and lipid peroxidation (55). The rise of this theme suggests that the field is increasingly trying to explain RA pathology not only in immunological terms, but also in terms of intracellular stress responses and cell-state susceptibility.

Another important observation is that thematic evolution in this field is characterized by coexistence rather than replacement. The temporal analysis of high-frequency keywords showed that long-standing terms such as rheumatoid arthritis, apoptosis, inflammation, and stromal-cell-related keywords remained active across broad time spans, whereas pyroptosis, ferroptosis, oxidative stress, and reactive oxygen species showed more recent concentration and stronger prominence in later years. This pattern indicates that the field has not abandoned its earlier concerns. Instead, it has entered a stage in which persistent core topics coexist with newly intensified mechanistic directions. Such coexistence is consistent with the thematic continuity and differentiation often observed in evolving research fields (56, 57). Rather than shifting abruptly from one paradigm to another, the field broadens its conceptual coverage while retaining a stable disease framework.

This interpretation is further strengthened by the correspondence analysis between keyword-derived thematic domains and reference co-citation clusters. The strongest correspondence values were concentrated in a limited number of domain-cluster pairs, such as inflammatory cell death pathways with Cluster 5, oxidative stress-related cell death with Cluster 4, and immune cell regulation with Cluster 3, whereas synovial fibroblast dysfunction showed stronger correspondence with Clusters 2 and 6. These relationships are particularly valuable because they demonstrate that the current thematic vocabulary of the field is structurally grounded in its intellectual base. In other words, the themes identified from author keywords are not superficial labels detached from the historical literature; they map onto specific knowledge clusters that have developed over time. This structural coupling between thematic domains and co-citation clusters suggests a relatively high degree of internal coherence within the field. It also supports the view that emerging hotspots such as ferroptosis and pyroptosis are being integrated into the existing RA research framework rather than simply appearing as transient, trend-driven topics (5, 41).

The source and disciplinary analyses add another important layer to this interpretation. The journal landscape was concentrated in rheumatology-, immunology-, and inflammation-related sources, whereas the dual-map overlay revealed strong citation flows among rheumatology and immunology, molecular biology and genetics, and clinical medicine. This pattern suggests that PCD research in RA is inherently interdisciplinary. Its core questions are disease-specific and clinically relevant, but its explanatory mechanisms are increasingly drawn from molecular cell death biology, redox biology, and systems-level mechanistic analysis (58–60). This cross-disciplinary structure likely contributes to the rapid expansion of the field, because it enables convergence between basic mechanistic studies and translational disease-oriented research (61, 62). The appearance of bioinformatics, machine learning, single-cell RNA sequencing, and WGCNA within the keyword structure also suggests that future progress will likely depend not only on identifying additional pathways, but also on improving cell-type-resolved and high-dimensional analytical approaches capable of clarifying how distinct death programs operate in different cellular compartments of the RA microenvironment (63–65).

Compared with these thematic and intellectual findings, the collaboration analyses mainly help contextualize the field’s development. Research output was concentrated in a limited number of highly productive countries, institutions, and authors, with China showing the largest publication volume. The additional country-level indicators further showed that publication output, citation impact, and international collaboration were not fully aligned. Although China contributed the largest number of publications, the United States had the highest total citations, while several countries with lower publication output, including Germany, the United Kingdom, the Netherlands, Switzerland, and Australia, showed relatively high MCP ratios. This pattern suggests that the field has developed through both concentrated national productivity and cross-national collaboration. A small number of productive research systems appear to provide continuity and volume, whereas collaborative ties may facilitate the exchange of methods, concepts, and emerging directions (66). However, from the perspective of scientific interpretation, these patterns are more informative about the organization of the research community than about the conceptual transformation of the field itself (67). For this reason, collaboration patterns are best understood as the structural background against which the more substantive changes in knowledge base and thematic evolution have occurred.

The sensitivity analyses further support the reliability of the main findings. The author keyword co-occurrence network retained the same five thematic domains across alternative occurrence thresholds, indicating that the thematic structure was not driven by a single threshold setting. The reference co-citation analysis showed that the core intellectual base was broadly stable across citation thresholds, although smaller or more recent clusters were more sensitive to threshold changes. This pattern is reasonable because emerging areas such as ferroptosis and oxidative stress have had less time to accumulate citations and may therefore be less stable under stricter citation thresholds. In addition, excluding the seven PubMed-only records did not alter the keyword co-occurrence or reference co-citation networks, suggesting that the small number of PubMed-only records did not materially affect the main conclusions.

Taken together, the findings of this study suggest that PCD has become an increasingly powerful framework for integrating multiple dimensions of RA pathogenesis (9). It connects classical synovial pathology with inflammatory amplification, links immune-cell regulation with stromal dysfunction, and bridges molecular cell death mechanisms with translational therapeutic questions (47, 68). The thematic expansion from apoptosis-centered studies to a broader landscape involving pyroptosis, ferroptosis, oxidative stress, extracellular vesicles, and emerging computational approaches indicates that the field is entering a more integrated stage of development. At this stage, the major challenge is no longer simply to identify additional forms of cell death relevant to RA, but to clarify how these death programs interact across cell types, signaling contexts, and disease stages, and how such interactions may be translated into more precise mechanistic models and therapeutic strategies (69).

### 4.3 Limitations and conclusion

Several limitations should be acknowledged. First, although the combined use of WoSCC and PubMed provided broad coverage of the literature, relevant publications indexed exclusively in other databases may not have been captured. WoSCC and PubMed also differ in bibliographic structure, although the final dataset was predominantly composed of WoSCC records and dataset-source sensitivity analysis showed that excluding the PubMed-only records did not alter the main keyword co-occurrence or reference co-citation results. Second, bibliometric results are affected by the quality of database metadata, particularly in relation to author-name disambiguation, institutional standardization, and keyword consistency. Third, citation-based indicators tend to favor established publications and may underrepresent very recent studies and newly emerging topics. This issue is particularly relevant to the terminal year and to emerging topics such as ferroptosis and oxidative stress, which have had less time to accumulate citations. Fourth, bibliometric analysis can reveal publication patterns, intellectual structures, and thematic associations, but it cannot establish biological causality, therapeutic efficacy, or clinical relevance. Despite these limitations, the present study provides a systematic and data-driven overview of the developmental trajectory, knowledge base, and thematic evolution of PCD research in RA.

In conclusion, research on programmed cell death in rheumatoid arthritis has progressed from a predominantly apoptosis-centered framework toward a broader and more differentiated mechanistic field. While classical RA pathogenesis remains the intellectual foundation, recent studies increasingly emphasize pyroptosis, ferroptosis, oxidative stress, inflammasome activation, and related translational directions. These findings indicate that multiple forms of regulated cell death are being increasingly incorporated into the broader pathophysiological model of RA, although further mechanistic and translational studies are needed to clarify their disease-specific roles.

## Data Availability

The datasets analyzed during the current study were derived from the Web of Science Core Collection and PubMed databases. The search strategies, eligibility criteria, and screening process are described in the Methods section. The bibliographic data underlying the findings of this study are publicly searchable through these databases, subject to their respective terms of use. The processed data used for analysis are available from the corresponding author on reasonable request.
