# Supplementary material for: Global research trends in programmed cell death in rheumatoid arthritis from 2001 to 2025: a bibliometric analysis
Source: Front Immunol. 2026 Jun 10;17:1837734. doi: 10.3389/fimmu.2026.1837734 (PMC13290722; doi:10.3389/fimmu.2026.1837734)
Supplement: Supplementary file 1 [file Table1.docx]

**Supplementary Table S1. Sensitivity analyses of network parameters and dataset source**

| **Sensitivity analysis** | **Main setting/dataset** | **Alternative setting/dataset** | **Items** | **Clusters** | **Links** | **TLS** | **Interpretation** |
| --- | --- | --- | --- | --- | --- | --- | --- |
| Author keyword co-occurrence | Occurrence ≥5 | Occurrence ≥4 | 326 | 5 | 3,420 | 8,830 | Main thematic domains retained |
| Author keyword co-occurrence | Occurrence ≥5 | Occurrence ≥5 | 242 | 5 | 2,781 | 7,995 | Main analysis |
| Author keyword co-occurrence | Occurrence ≥5 | Occurrence ≥6 | 195 | 5 | 2,389 | 7,467 | Main thematic domains retained |
| Reference co-citation | Citation ≥20 | Citation ≥18 | 354 | 5 | 19,242 | 46,651 | Core intellectual domains retained; smaller clusters partially merged |
| Reference co-citation | Citation ≥20 | Citation ≥20 | 298 | 6 | 15,201 | 39,118 | Main analysis |
| Reference co-citation | Citation ≥20 | Citation ≥22 | 238 | 5 | 11,099 | 31,245 | Core intellectual domains retained; smaller or emerging domains less independent |
| Dataset-source sensitivity, author keywords | Full dataset, n=3,168 | WoSCC-only, n=3,161 | 242 | 5 | 2,781 | 7,995 | Unchanged after excluding seven PubMed-only records |
| Dataset-source sensitivity, reference co-citation | Full dataset, n=3,168 | WoSCC-only, n=3,161 | 298 | 6 | 15,201 | 39,118 | Unchanged after excluding seven PubMed-only records |

**Note**: Sensitivity analyses were performed to assess the robustness of the main network-based findings. For author keyword co-occurrence analysis, alternative minimum occurrence thresholds of 4 and 6 were compared with the main threshold of 5. For reference co-citation analysis, alternative minimum citation thresholds of 18 and 22 were compared with the main threshold of 20. Dataset-source sensitivity analysis was performed by excluding the seven PubMed-only records and repeating the author keyword co-occurrence and reference co-citation analyses using the same settings. “Items” indicates the number of nodes included in each network. “Links” indicates the number of connections between nodes. TLS, total link strength.

**Supplementary Table S2. Country-level productivity, citation impact, and collaboration indicators**

| **Rank** | **Country/region** | **Overall publications** | **Total citations** | **Average citations** | **SCP** | **MCP** | **MCP ratio** |
| --- | --- | --- | --- | --- | --- | --- | --- |
| 1 | China | 1,507 | 34,518 | 22.91 | 1,386 | 121 | 0.080 |
| 2 | United States | 531 | 53,521 | 100.79 | 284 | 247 | 0.465 |
| 3 | Japan | 207 | 9,977 | 48.20 | 157 | 50 | 0.242 |
| 4 | Germany | 146 | 17,586 | 120.45 | 65 | 81 | 0.555 |
| 5 | South Korea | 137 | 6,505 | 47.48 | 108 | 29 | 0.212 |
| 6 | United Kingdom | 135 | 20,499 | 151.84 | 55 | 80 | 0.593 |
| 7 | India | 105 | 3,121 | 29.72 | 70 | 35 | 0.333 |
| 8 | Italy | 95 | 4,533 | 47.72 | 58 | 37 | 0.389 |
| 9 | France | 89 | 6,137 | 68.96 | 52 | 37 | 0.416 |
| 10 | Netherlands | 74 | 5,245 | 70.88 | 24 | 50 | 0.676 |
| 11 | Switzerland | 71 | 5,091 | 71.70 | 25 | 46 | 0.648 |
| 12 | Australia | 51 | 3,230 | 63.33 | 22 | 29 | 0.569 |
| 13 | Canada | 45 | 2,520 | 56.00 | 26 | 19 | 0.422 |
| 14 | Iran | 45 | 1,529 | 33.98 | 29 | 16 | 0.356 |
| 15 | Spain | 44 | 3,093 | 70.30 | 32 | 12 | 0.273 |

**Note**: Countries/regions were ranked by overall publication output. Overall publications indicate the number of publications involving at least one author from the corresponding country or region. Total citations indicate the total citation counts of publications involving each country/region. Average citations were calculated as total citations divided by overall publications. SCP denotes single-country publications, and MCP denotes multiple-country publications. MCP ratio was calculated as MCP divided by overall publications.

**Supplementary Table S3. Bridging references linking classical RA pathology with emerging programmed cell death mechanisms**

| **Reference** | **Cluster** | **CC** | **TLS** | **Link to C1–C2** | **Classical RA domain** | **Emerging PCD domain** | **Bridging role** |
| --- | --- | --- | --- | --- | --- | --- | --- |
| Wu J, 2022, Nature Communications | 4 | 69 | 596 | 279 | RA inflammatory pathogenesis and immune-mediated joint damage | Ferroptosis / regulated cell death | A highly connected Cluster 4 reference linking emerging ferroptosis-related mechanisms with the broader RA pathogenesis cluster |
| Dixon SJ, 2012, Cell | 4 | 66 | 487 | 189 | Conceptual basis for interpreting oxidative injury in inflammatory disease | Ferroptosis | Provides the foundational definition of ferroptosis and supports the incorporation of ferroptotic cell death into RA-related mechanistic interpretations |
| Zhao T, 2022, Frontiers in Immunology | 4 | 50 | 426 | 212 | RA immune-inflammatory microenvironment | Ferroptosis-related immune regulation | Connects ferroptosis with immunological mechanisms relevant to RA and shows strong structural linkage with classical RA clusters |
| Phull AR, 2018, Chemico-Biological Interactions | 4 | 40 | 322 | 201 | Inflammation and oxidative injury in arthritis-related contexts | Oxidative stress / redox regulation | Bridges inflammatory joint pathology with redox-mediated cellular damage and therapeutic exploration |
| Ling HZ, 2022, Rheumatology | 4 | 39 | 376 | 126 | RA synovial pathology and inflammatory activity | Ferroptosis / oxidative stress | Links RA disease mechanisms with ferroptosis-related molecular changes |
| Xie ZX, 2021, Inflammation | 4 | 35 | 419 | 181 | Inflammatory response in RA-related pathology | Ferroptosis / inflammatory regulation | Shows strong co-citation linkage between inflammatory RA mechanisms and emerging ferroptosis-related research |
| Luo HS, 2021, Experimental and Therapeutic Medicine | 4 | 34 | 352 | 138 | RA inflammatory injury and therapeutic context | Oxidative stress / cell death regulation | Connects oxidative-stress-related injury with RA-associated therapeutic mechanisms |
| Jiang XJ, 2021, Nature Reviews Molecular Cell Biology | 4 | 33 | 291 | 117 | General disease-mechanism framework | Ferroptosis biology | Provides an updated mechanistic framework for ferroptosis, facilitating interpretation of ferroptosis within RA pathogenesis |
| Zhou RP, 2022, Redox Biology | 4 | 28 | 289 | 117 | Inflammation-related redox imbalance | Ferroptosis / oxidative stress | Bridges redox biology, lipid peroxidation, and regulated cell death in inflammatory disease contexts |
| Hitchon CA, 2004, Arthritis Research & Therapy | 4 | 26 | 171 | 126 | Oxidative damage in RA | Oxidative stress | Serves as an earlier RA-specific oxidative-stress reference connecting classical RA pathology with later ferroptosis/redox-related research |
| Mateen S, 2016, PLOS ONE | 4 | 26 | 228 | 111 | RA inflammatory and oxidative pathology | Oxidative stress / antioxidant response | Links oxidative stress and inflammatory mechanisms in RA-relevant experimental contexts |
| Stockwell BR, 2017, Cell | 4 | 27 | 243 | 103 | Conceptual framework for disease-related cell death | Ferroptosis | Provides a broader ferroptosis framework that supports later disease-specific applications in RA |

**Note**: Bridging references were selected from Cluster 4 of the reference co-citation network according to co-citation count, total link strength, cross-cluster link strength with Clusters 1–2, and thematic relevance. Cluster refers to the co-citation cluster shown in Table 1. CC denotes co-citation count, and TLS denotes total link strength. Link to C1–C2 indicates the total co-citation link strength between each listed Cluster 4 reference and references in Clusters 1 and 2. C1 represents the general pathogenesis and conceptual framework of RA, and C2 represents classical RA mechanisms, synovial pathology, and inflammatory signaling. RA, rheumatoid arthritis; PCD, programmed cell death.
